# Duloxetine use in chronic painful conditions – individual patient data responder analysis

**DOI:** 10.1002/j.1532-2149.2013.00341.x

**Published:** 2013-06-03

**Authors:** RA Moore, N Cai, V Skljarevski, T R Tölle

**Affiliations:** 1 Pain Research and Nuffield Division of Anaesthetics, University of OxfordUK; 2 Lilly Corporate Center, Eli Lilly and CompanyIndianapolis, USA; 3 Klinik für Neurologie, Technische Universität MünchenGermany

## Abstract

**Background:**

Duloxetine has been studied in four distinct chronic pain conditions – osteoarthritis (OA), fibromyalgia, chronic low back pain (CLBP) and diabetic peripheral neuropathic pain (DPNP). These trials have involved large numbers of patients with at least moderate pain, and have used similar methods for recording pain intensity, over about 12 weeks.

**Methods:**

Data from the trials were pooled according to painful condition, and reanalysed at the level of the individual patient and using increasing levels of pain intensity reduction (<15%, 15–29%, 30–49%, ≥50%), with different imputation methods on withdrawal.

**Results:**

The proportion of patients recording at least 50% pain intensity reduction plateaued after 2–6 weeks in fibromyalgia, and 8–12 weeks in other conditions. The duloxetine-specific benefit [number needed to treat (NNT) for at least 50% pain intensity reduction] was fairly constant after about 2 weeks for DPNP and fibromyalgia and after about 4 or 5 weeks for OA and CLBP. In all conditions, responses were bimodal, with patients generally experiencing either very good or very poor pain relief. Last-observation-carried-forward imputation produced numerically and occasionally statistically better (lower) NNTs than use of baseline-observation-carried-forward (true response).

**Conclusions:**

Baseline-observation-carried-forward (true response), which combines the success of high levels of pain relief with the failure to experience pain relief on withdrawal of the drug is conservative and probably reflective of clinical practice experience. The distribution of effect was not normal; few patients had the average response and averages are not an appropriate descriptor for these data.

**What's already known about this topic?:**

- Last-observation-carried-forward (LOCF) imputation overestimates efficacy when adverse event withdrawals are high.
- Previous analyses of duloxetine in four chronic pain conditions reported mainly average pain changes using LOCF imputation.

**What does this study add?:**

- Responses were bimodal: patients generally experienced very good or very poor pain relief. Last-observation-carried-forward results were numerically lower (better) than true responder.
- Duloxetine was an effective analgesic in all four chronic pain conditions using highest level of evidence.

## 1. Introduction

The recognition of a number of potential biases in clinical trials and meta-analyses in chronic pain has led to the adoption of new standards of evidence (Moore et al., 2010a). These include using long duration studies (longer than 8 weeks), establishing a threshold for substantial benefit, typically that of at least 50% pain intensity reduction (Dworkin et al., 2008), and avoiding potential bias from imputation methods by using a true responder definition, whereby withdrawal for any reason is considered non-response (Moore et al., 2012b). Part of the impetus behind these changes is the increasing recognition that a favourable response to treatment (high level of pain relief) is associated with improved sleep, less fatigue and depression, better function and ability to work, and higher quality of life; without substantial pain relief, these improvements are not seen (Hoffman et al., 2010; Moore et al., 2010e; Straube et al., 2011).

A National Academy of Sciences report (Panel on Handling Missing Data, 2010) recommended that imputation methods, such as last-observation-carried-forward (LOCF) and baseline observation carried forward (BOCF), should not be used as the primary approach to the treatment of missing data unless assumptions underlying them are scientifically justified. LOCF methods have been commonly used in analysing pain trial data where they may prove useful for group mean analyses. However, when a large percentage of patients withdraw because of lack of efficacy or adverse events (Moore et al., 2010d), as is the case particularly with opioid use (Moore et al., 2012b), a BOCF approach is warranted. In the case of chronic pain, the definition of a true responder as a patient with a high level of pain relief sustained for 12 weeks without intolerable adverse events (essentially a BOCF approach) is of practical clinical value.

We have limited knowledge of how true responder (BOCF) analyses impact estimates of treatment efficacy across chronic pain conditions, although an analysis of the effect of different imputation methods has been published for duloxetine in chronic low back pain (CLBP) trials (Liu-Seifert et al., 2010). True responder analyses have been conducted for a number of chronic pain conditions, including osteoarthritis (OA), CLBP and ankylosing spondylitis with etoricoxib (Moore et al., 2010a, 2010c; Peloso et al., 2011), and fibromyalgia with pregabalin (Straube et al., 2010). There are only two known reports of true responder analyses for neuropathic pain, one from a pooled analysis of pregabalin in several neuropathic pain conditions (Semel et al., 2010) and a small trial in diabetic peripheral neuropathic pain (DPNP; Hewitt et al., 2011).

Here we report the responder analysis findings for a single serotonin-norepinephrine reuptake inhibitor (SNRI), duloxetine, in four distinct chronic pain conditions, OA, fibromyalgia, CLBP and DPNP. To the best of our knowledge, this is the first patient level analysis of an intervention assessed using important patient-centred pain outcomes and across four different chronic pain conditions. We report on speed of onset of effect, different levels of pain relief, and the effect of imputation method (LOCF and BOCF) on measures of treatment efficacy.

## 2. Methods

Data for these analyses were from duloxetine randomized trials (Table 1), conducted in OA [studies HMFG, HMEP and HMGL (Chappell et al., 2009, 2011; Frakes et al., 2011], fibromyalgia (studies HMBO, HMCA, HMCJ and HMEF (Arnold et al., 2004, 2005; Chappell et al., 2008; Russell et al., 2008); ], CLBP (studies HMEN, HMEO and HMGC (Skljarevski et al., 2009; 2010a, 2010b)), and DPNP [studies HMVAa, HMVAb and HMAW (Goldstein et al., 2005; Raskin et al., 2005; Wernicke et al., 2006); ]. All were randomized, double blind, placebo-controlled studies, conducted in patients with at least moderate initial pain intensity (≥ 4 on a 0–10 numerical rating scale). Primary endpoints were measured at 12–15 weeks, apart from one study in OA where the primary endpoint was at 8 weeks. We used data from placebo and duloxetine doses of either 60 mg or 120 mg, and pooled data from different doses (60 mg and 120 mg) or dosing schedules, since there appear to be no differences in efficacy with various dosing regimens (Sultan et al., 2008; Lunn et al., 2009; Bradley et al., 2010).

**Table 1:** Data sources for analyses

Chronic pain condition	Study identifier	Length of acute phase of study (weeks)	Dosing schedule	Pain measures		Primary endpoint (timepoint)
				Weekly mean of the daily 24-h average pain severity on a 11-point Likert scale	BPI (weeks)	
Osteoarthritis	HMFG	13	60 Week 7: increased to120 based on reduction in BPI average pain score	Yes	4, 7, 13	BPI 24-h average pain (13 weeks)
	HMEP	13	60 Week 7 randomized to 60 or 120	Yes	4,7,13	Weekly mean of 24-h average pain (13 weeks)
	HMGL	10	60 Week 3: increased to120 based on weekly mean of average pain score	Yes	3,4,8,10	Weekly mean of 24-h average pain, measured at 8 weeks
Fibromyalgia	HMBO	12	120	No	1,2,4,6,8,10,12	Fibromyalgia Impact Questionnaire (FIQ) pain item and the total FIQ scores (12 weeks)
	HMCA	12	60, 120	No	1,2,4,6,8,10,12	BPI 24-h average pain (12 weeks)
	HMCJ	15	20, 60, 120	No	1,2,4,7,11,15	BPI 24-h average pain and PGI-Improvement (15 weeks)
	HMEF	27	60 Week 13: increased to120 based on reduction in BPI average pain score.	No	1,2,4,6,8,13,18,23,27	BPI 24-h average pain and PGI-Improvement (13 weeks)
Chronic low back pain	HMEN	13	60 Week 7: increased to 120 based on BPI	Yes	4,7,13	BPI 24-h average pain (13 weeks)
	HMEO	13	20, 60, 120	Yes	4,7,13	Weekly mean of the 24-h average pain (13 weeks)
	HMGC	12	60	Yes	3,6,9,12	BPI 24-h average pain (12 weeks)
Diabetic painful neuropathic pain	HMAVa	12	60, 120	Yes	4,8,12	Weekly mean of the 24-h average pain (12 weeks)
	HMAVb	12	60, 120	Yes	4,8,12	Weekly mean of the 24-h average pain (12 weeks)
	HMAW	12	20, 60, 120	Yes	1,2,3,4,6,8,10,12	Weekly mean of the 24-h average pain (12 weeks)

BPI, British Pain Inventory.

For analysis of time course response we used the weekly mean of average pain from daily diary records for the OA, CLBP, and DPNP datasets, and British Pain Inventory average pain for the fibromyalgia datasets. The outcome used was at least 50% pain intensity reduction using BOCF.

For the comparison of LOCF and BOCF imputation methods we used the primary endpoints of the individual studies (pain, measured on a 10-point numerical rating scale, see Table 1), except in the case of Study HMBO. In all except the fibromyalgia studies the scale was administered both using a diary and at scheduled office visits; for fibromyalgia, the scale was administered only at scheduled office visits.

For the responder analyses, a responder was defined as a patient demonstrating a specified improvement in pain level, defined as at least 15, 30, 50 or 70% pain intensity reduction at the primary endpoint compared with baseline; these cutpoints have been used previously (Barthel et al., 2010; Moore et al., 2010b, 2010c; Straube et al., 2010), and the 30 and 50% cutpoints have been used to define moderate and substantial benefits (Dworkin et al., 2008). We also recorded responders with <15%, 15–29%, 30–49% and ≥50% pain intensity reduction. Withdrawal from treatment for any reason was regarded as non-response and equivalent to BOCF, since imputation with the baseline level of pain intensity would exclude achievement of any of these levels of response. Responders were therefore considered true responders. We also calculated responders using LOCF imputation, where the last non-missing observation was carried forward from the time of withdrawal to the end of the trial.

In the analyses, we used the intention-to-treat definition as patients randomized and having received at least one dose of duloxetine or placebo; there were 4343 patients who were randomized and who had a baseline pain value (OA, *n* = 1011; fibromyalgia, *n* = 1332; CLBP, *n* = 982; DPNP, *n* = 1024) included in the responder analyses. Of these, 4238 (98%) had at least one pain recording after taking the first dose; because imputation is not possible without at least one post-treatment pain recording, this number was used as the denominator for the LOCF imputation analyses. Analyses were conducted according to painful condition, as different painful conditions can have different levels of response to placebo and active treatment (Moore et al., 2009). We calculated the number and percentage of responders by level of response for each treatment and time point and the number needed to treat (NNT) compared with placebo [with 95% confidence interval (CI; Cook and Sackett, 1995) ]. The relative risk or benefit with 95% CI was calculated using the fixed effects model (Morris and Gardner, 1995) and considered statistically significant when the 95% CI did not include 1. NNT values were only calculated where there was a statistically significant difference between active and placebo treatments. Statistically significant differences between NNTs comparing different drugs or doses were calculated using the *z*-test (Tramer et al., 1997), using data only from trials in which items being compared were both used.

Effects of excess adverse event withdrawal on percentage overestimation of NNT for LOCF imputation over true responder definition were calculated for duloxetine 60/120 mg for each chronic painful condition. The magnitude of the overestimation of treatment effect with LOCF (that is, the NNT increase defining withdrawal as nonresponse (BOCF) compared with use of LOCF imputation) was expressed as a percentage [(100 × BOCF/LOCF imputation) − 100] (Moore et al., 2012b).

## 3. Results

### 3.1 Time course of response

**Fig 1:**
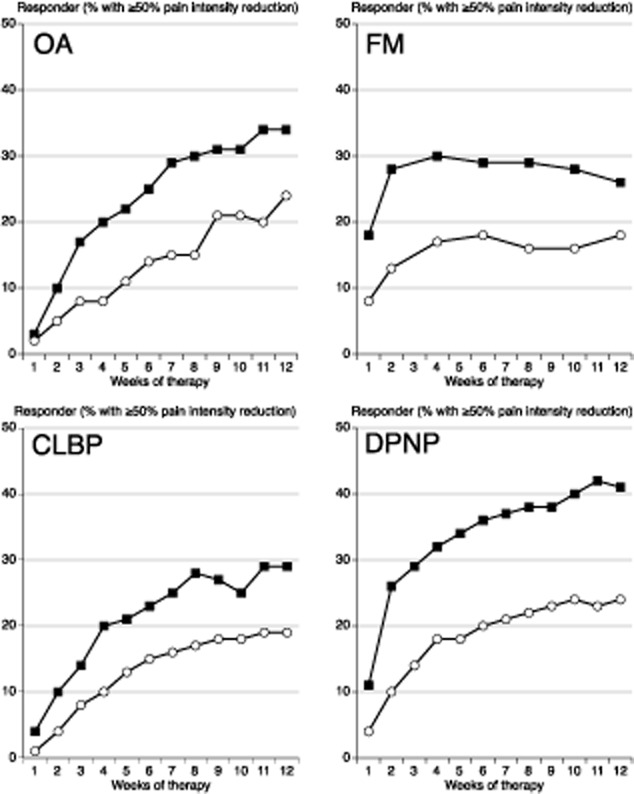
Percentage of patients achieving at least 50% pain intensity reduction with duloxetine 60/120 mg (black symbol) and placebo (white symbol) in four chronic pain conditions.

Fig. 1 shows the proportion of patients with at least 50% pain intensity reduction using BOCF (true responders) over 12 weeks in each of the conditions. This increased over time in all four painful conditions. For OA, CLBP and DPNP the proportion of patients with at least 50% pain intensity reduction reached 30–40% with duloxetine and 18–25% with placebo by week 12. For fibromyalgia, response rates with duloxetine reached 28% by week 2 with duloxetine, and 18% by week 6 with placebo.

**Fig 2:**
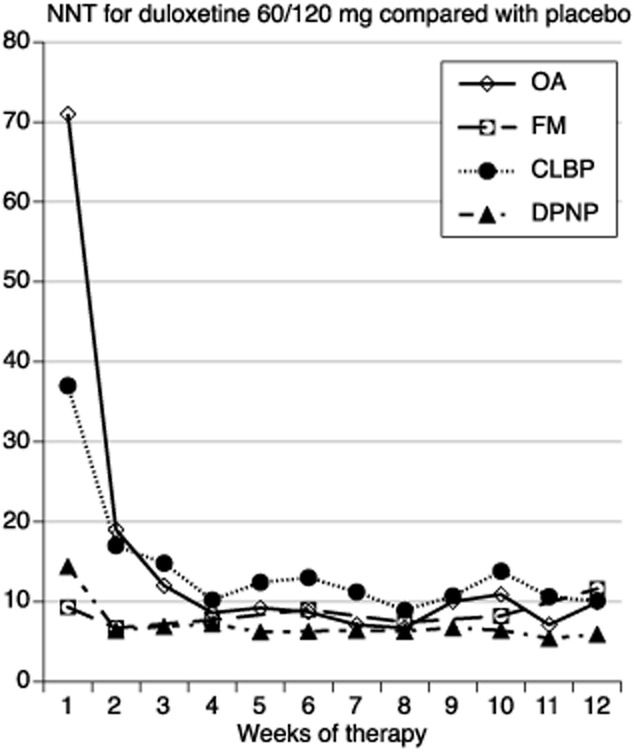
NumberS needed to treat calculated over 12 weeks for at least 50% pain intensity reduction with duloxetine 60/120 mg compared with placebo.

The time course of efficacy in the four painful conditions was somewhat different. For the outcomes of at least 50% pain intensity reduction for true responders, Fig. 2 demonstrates that consistent NNT values were achieved within 2 weeks for DPNP and fibromyalgia, and by weeks 4 or 5 for the chronic musculoskeletal conditions of OA and CLBP.

### 3.2 Extent of response and effect of LOCF imputation

Table 2 shows the degree of response at ≥15%, ≥30%, ≥50% and ≥70% pain intensity reduction by the end of trial (8–12 weeks) for duloxetine 60/120 mg and placebo. The NNTs calculated at these different levels of response were typically large at very low and very high levels of response (Table 2), and lower with response levels of at least 30% or at least 50% pain intensity reduction. This was the case whether true responses or LOCF imputation was used. However, NNTs calculated using the true responder definition were always numerically higher than those calculated using LOCF imputation, and in one of the 16 cases statistically higher.

**Table 2:**
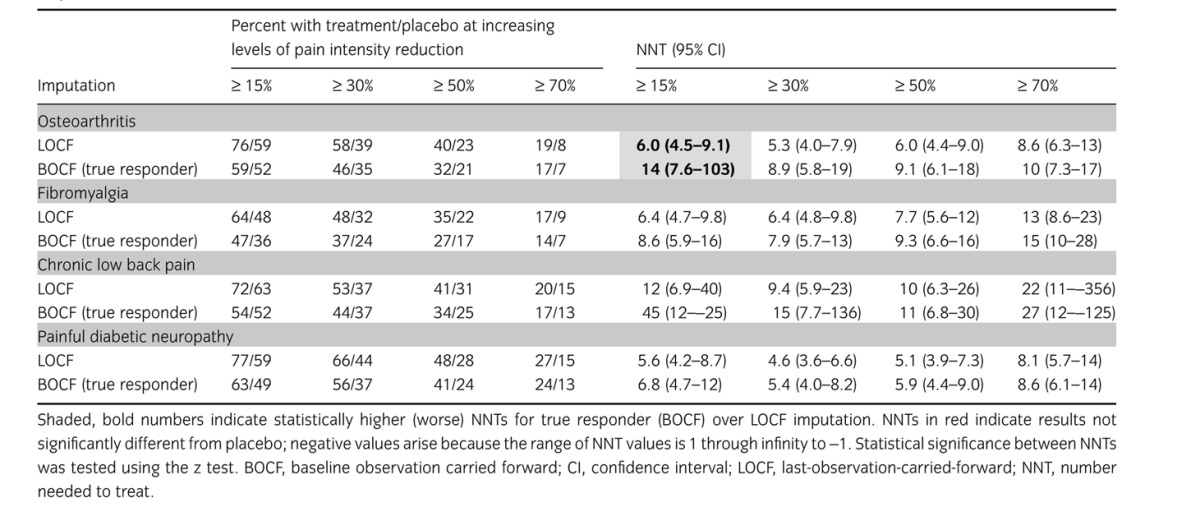
Effect of response level and imputation on percentage of responders with duloxetine 60/120 mg and placebo, and NNTs calculated from responder rates

**Fig 3:**
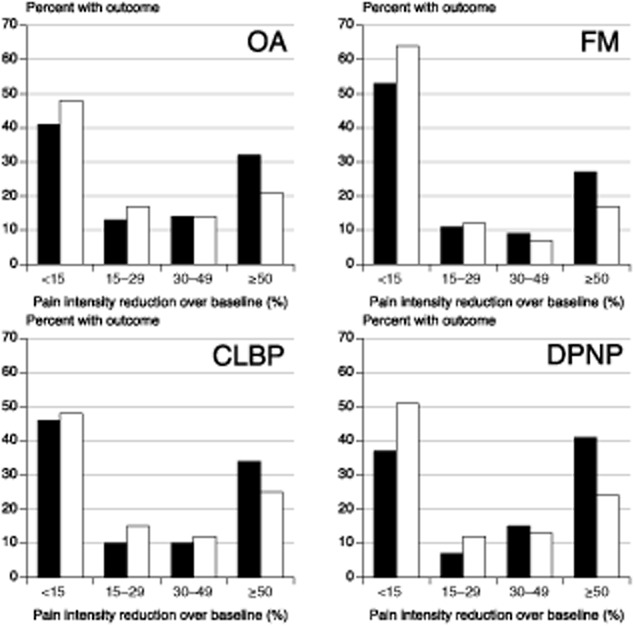
Patterns of pain intensity reduction for four different painful conditions using baseline observation carried forward true responders for duloxetine 60/120 mg (black symbol) and placebo (white symbol). Average pain intensity reductions with standard deviation were: osteoarthritis – duloxetine 32 ± 32%, placebo 23 ± 28%; fibromylagia – duloxetine 24 ± 35%, placebo 15 ± 30%; chronic low back pain – duloxetine 30 ± 35%, placebo 24 ± 35%; painful peripheral diabetic neuropathy – duloxetine 38 ± 37%, placebo 23 ± 35%.

Fig. 3 shows the distribution of response for each of the four pain conditions using BOCF true responders and intermediate levels of response. For each condition the distribution was distinctly non-Gaussian. With duloxetine or placebo, the majority of patients had either a very good response (≥50% pain intensity reduction) or poor response (<15% pain intensity reduction); few had intermediate levels of pain intensity reduction. Average pain intensity reductions ranged between 24% and 38% with duloxetine, and 15% and 24% with placebo; the average reflected the experience of only a small proportion of patients.

### 3.3 Effect of withdrawal rates on overestimation of NNT with LOCF imputation

Table 3 shows withdrawals from treatment because of adverse events and lack of efficacy in each of the four chronic painful conditions. There were clear differences in withdrawal rate patterns between fibromyalgia and the other three conditions. For placebo, adverse event and lack of efficacy withdrawals were low (<7.5% and <4%, respectively) in DPNP, OA and CLBP studies, but higher (>10%, >11%) in fibromyalgia studies. Adverse event withdrawals with duloxetine were higher than with placebo (>14%), but lack of efficacy withdrawals lower in each condition.

**Table 3:** Study withdrawal with duloxetine 60/120 mg and placebo in four painful conditions

Condition			Placebo		Duloxetine	
	Number of		Percent withdrawals due to		Percent withdrawals due to	
	Trials	Patients	Adverse events	Lack of efficacy	Adverse events	Lack of efficacy
Osteoarthritis	3	1011	7.3	3.2	15.7	1.0
Fibromyalgia	4	1332	10.5	11.8	18.4	5.5
Chronic low back pain	3	982	6.4	3.6	16.7	1.9
Painful diabetic neuropathy	3	1024	5.0	2.9	13.6	1.0

Previous research has shown that an excess of adverse event withdrawals is the major driver for overestimation of treatment effect with LOCF compared with BOCF (Moore et al., 2012b) For each of these four chronic pain conditions, the relationship between adverse event withdrawals with active drug over placebo and overestimation of treatment effect with LOCF imputation over BOCF true responders was consistent with that relationship.

## 4. Discussion

To the best of our knowledge, this is the first patient-level analysis of a single intervention (duloxetine 60/120 mg) assessed using important patient-centred pain outcomes and across four different chronic pain conditions, spanning neuropathic pain, musculoskeletal pain and fibromyalgia. The strength of the analysis is that the data come from well-conducted randomized, double-blind trials, of trial duration longer than 8 weeks, and with large enough treatment arms to limit some known biases, such as sample size (Nüesch et al., 2010). The analysis met all the criteria for reporting unbiased data in chronic pain (Moore et al., 2010a), and the effect of potentially large biases from imputation method could be examined and eliminated (Moore et al., 2012b).

The key findings from this analysis were that:

1. The proportion of patients recording at least 50% pain intensity reduction plateaued after 2–6 weeks for fibromyalgia, and 8–12 weeks for the other conditions (Fig. 1).
2. The duloxetine-specific benefit (NNT for at least 50% pain intensity reduction) was fairly constant after about 2 weeks for DPNP and fibromyalgia, and after about 4 or 5 weeks for OA and CLBP (Fig. 2).
3. In all conditions, responses were bimodal, with patients generally experiencing either very good or very poor pain relief (Fig. 3).
4. LOCF imputation produced numerically and sometimes statistically better (lower) NNTs than use of BOCF (true response); the latter combines both the success of high levels of pain relief with the failure to experience pain relief on withdrawal of the drug for any reason and is probably more reflective of clinical practice experience (Moore et al., 2012b).

It is instructive to compare these results for duloxetine with what is known about other drugs used in chronic pain conditions, despite the lack of any comprehensive background information in any individual painful condition. For example, findings have been published on pain relief with non-steroidal anti-inflammatory drugs (NSAIDs) in OA (Moore et al., 2010b), CLBP (Moore et al., 2010c), and ankylosing spondylitis (Peloso et al., 2011), as well as pregabalin in fibromyalgia (Straube et al., 2011); in all cases, proportion of responders remained reasonably constant after about 2 weeks of treatment. These earlier findings are similar to those seen here with duloxetine use in fibromyalgia (Fig. 1), but are in contrast to those seen with duloxetine in DPNP, OA and CLBP, where the percentage of responders rose consistently throughout the trial periods. Given our current experience and knowledge, it is not possible to determine whether the differences are a feature of the painful condition, the nature of responses with SNRIs, or some otherwise-overlooked feature of the way the trials were conducted. More analyses like this for duloxetine are needed to fully understand time courses of action of different drugs in chronic pain.

Previous work has also demonstrated a tendency for NNTs to increase over a 12-week study period, sometimes quite substantially, with NSAID use in CLBP (Moore et al., 2010c), with ibuprofen, but not other NSAID, use in OA (Moore et al., 2010b), and with pregabalin use in fibromyalgia (Straube et al., 2011). For duloxetine, by contrast, NNTs fell during the first 2–5 weeks of treatment, and were consistent thereafter in all four conditions, much as has been reported with NSAID use in ankylosing spondylitis (Peloso et al., 2011). Again, different circumstances seem to deliver different patterns of response.

What is consistent between the current findings and those from previous analyses is the bimodal distribution of response by the end of trial, with the majority of patients having either a substantial level of pain relief or very little; few experienced the ‘average’ result. Averages are not appropriate descriptors for these data, especially when the standard deviation of the average response is of the same magnitude as, or even larger than, the average, as here (Fig. 3).

The consistency of the bimodal distribution is remarkable, in this study over four different chronic pain conditions for duloxetine, and generally in other studies across different pharmacological interventions in acute pain (Moore et al., 2011), NSAIDs in musculoskeletal conditions (Moore et al., 2010b, 2010c; Peloso et al., 2011) and amitriptyline in neuropathic pain (Moore et al., 2012a). It emphasizes most strongly that currently available analgesics can deliver substantial benefits in pain relief (≥50% pain intensity reduction) for a minority of patients with chronic pain, and this level of pain relief is known to be accompanied by large improvements in sleep, depression, fatigue, function, quality of life and ability to work (Hoffman et al., 2010; Moore et al., 2010e; Straube et al., 2011).

Another feature where the results of this analysis are generally in keeping with those reported for other drugs is in the relationship between NNT calculated using either BOCF or LOCF and the degree of pain intensity reduction. The finding of lower (better) NNTs with the 30% and 50% cutpoints compared with lower or higher cutpoints is similar to the findings with etoricoxib in OA and ankylosing spondylitis (Moore et al., 2010b; Peloso et al., 2011), and with pregabalin in fibromyalgia (Straube et al., 2010). This indicates that the outcomes defined as moderate and substantial benefit to patients (Dworkin et al., 2008) are those with the greatest sensitivity to distinguish efficacy, similar to acute pain (Moore et al., 2011).

What we have, then, are consistent findings across chronic pain studies for the bimodal distribution of pain response and an overestimation of treatment effect in the presence of high adverse event withdrawals with active drug. This has a potentially major impact on the development of guidelines unless all potential sources of bias are considered so that like is only compared with like. What we do not have is consistency in terms of time to response or evolution of efficacy, which seem to differ between conditions and drugs; this may be the type of drug, or the condition, or an interaction between both. We do not know enough to be sure.

This analysis of responder rates of duloxetine in four different chronic pain conditions is possibly unique, and has the potential to induce a radical shift in everyday chronic pain treatment. Currently available medications for symptomatic treatment of chronic pain are traditionally grouped into different medication categories – NSAIDs, opioids, anticonvulsants and antidepressants. These categories are reflections of either the pathophysiological concept behind the particular pain category (e.g. nociceptive vs. inflammatory vs. neuropathic) or where these treatments were first developed. Chronic pain treatments have often ‘borrowed’ drugs developed in other areas as much as using therapies developed on a mechanistic basis.

If we could identify individual patients with any pain condition in whom specific biological mechanisms were responsible for their pain (nociceptive, inflammatory, neuropathic or some other unknown mechanism), individualized treatment specifically targeting those mechanisms might be employed. Any practical method of ascertaining which mechanism was responsible for an individual patient's pain (Jensen and Baron, 2003; Tölle and Backonja, 2013) would demand a paradigm shift over current clinical practice of trying different drugs in individual patients to find one that worked. In this context, not surprisingly, a single drug can have substantial effects in many different chronic pain conditions, if the appropriate mechanism, e.g. central sensitization or imbalanced descending control, is involved and properly met by the drug. These theoretical considerations together with clinical observations open up a much broader and favourable treatment regimen than currently typically practiced.

Thoughtful analyses have demonstrated similar results for pharmacological interventions in chronic pain, which is that at best half of patients treated with any single drug obtains ‘moderate’ pain relief (Dworkin et al., 2010; Finnerup et al., 2010), although this proportion falls when higher levels of pain relief and evidence standards are applied (Moore et al., 2010a, 2012b). In general, though, drugs tend to have broadly equivalent efficacy in particular conditions, as is seen with duloxetine and amitriptyline (Kaur et al., 2011), or pregabalin and amitriptyline (Bansal et al., 2009). Importantly, this broad similarity in overall efficacy does not exclude individual patients responding to one drug but not another, as with amitriptyline and nortriptyline (Watson et al., 1998), or preferring one drug over another, as with duloxetine and amitriptyline (Kaur et al., 2011). Drugs may have equal efficacy on average, but not in individuals.

## 5. Conclusion

This analysis confirms several key elements of evidence regarding our understanding and use of clinical trial data to support clinical practice. LOCF probably consistently overestimates the beneficial effects of treatment, and studies of short duration may provide inaccurate results. A true responder analysis of data from studies of longer duration (6–8 or more weeks), and using the ideal outcome of at least 50% pain intensity reduction are needed before we can be confident of making appropriate indirect comparisons between treatments, especially in guideline development. Last but not least, the clinical data show that the current concept of using certain drug classes for specific chronic pain states should be abandoned.

### Author contributions

RAM and VS developed the original idea for the study and the broad aims and objectives. NC and RAM performed the analyses. TT provided clinical insights. RAM and VS developed the original draft, and all authors contributed to the development of interim and final drafts.
